# Exploring the impact of word-of-mouth about Physicians’ service quality on patient choice based on online health communities

**DOI:** 10.1186/s12911-016-0386-0

**Published:** 2016-11-26

**Authors:** Naiji Lu, Hong Wu

**Affiliations:** School of Medicine and Health Management, Tongji Medical College, Huazhong University of Science and Technology, Wuhan, People’s Republic of China

**Keywords:** Online health communities, Word-of-mouth, Service quality, Technical quality, Functional quality, Patient choice, Disease risk

## Abstract

**Background:**

Health care service is a high-credence service and patients may face difficulties ascertaining service quality in order to make choices about their available treatment options. Online health communities (OHCs) provide a convenient channel for patients to search for physicians’ information, such as Word-of-Mouth (WOM), particularly on physicians’ service quality evaluated by other patients. Existing studies from other service domains have proved that WOM impacts consumer choice. However, how patients make a choice based on physicians’ WOM has not been studied, particularly with reference to different patient characteristics and by using real data.

**Methods:**

One thousand eight hundred fifty three physicians’ real data were collected from a Chinese online health community. The data were analyzed using ordinary least squares (OLS) method.

**Results:**

The study found that functional quality negatively moderated the relationship between technical quality and patient choice, and disease risk moderated the relationship between physicians’ service quality and patient choice.

**Conclusions:**

Our study recommends that hospital managers need to consider the roles of both technical quality and functional quality seriously. Physicians should improve their medical skills and bedside manners based on the severity and type of disease to provide better service.

## Background

With the development of Web 2.0 technologies, social media provides a unique platform for products and services to be evaluated by their purchasers and users. A large number of online websites have already provided consumer review forums to facilitate word-of-mouth (WOM) communication. Online health communities such as Haodf.com (http://www.haodf.com/) and Guahao.com (http://www.guahao.com/) also provide the WOM functions, by which patients can get information about the quality of the health services provided by physicians.

High-credence services are intangible in nature and in general associated with a high degree of uncertainty because most customers lack the professional knowledge to assess the service quality [1]. It’s hard to evaluate as it depends on the ability of the service provider to communicate effectively. Since Health services contain high degrees of uncertainty and risk, they are considered to be high-credence services [2]. It can be difficult for patients to evaluate physicians’ services.

Before the emergence of OHCs, it is lack of channels for patients to access physicians’ health quality. It’s hard to get the evaluations of health quality for each physician, so prior studies mainly focus on the Evaluations of health quality have mainly focused on institutions such as hospitals or nursing homes (organizational level) based on questionnaire survey [3]. Contrastingly, there is limited information that patient can access regarding individual physicians’ service quality (individual level) in the public domain. Due to the lack of other channels for patients’ to access physicians’ service quality information, online physician ratings are becoming increasingly valuable [4]. Online health communities (OHCs) provide a new kind of health service in recent years in China, so there are still few research based on OHCs. Since the development of online health communities, Word-of-Mouth (WOM) review systems help us to explore the relationship between health quality of physicians and patient choice. Internet ratings of physicians have received extensive attention from both the media and other physicians [5]. As a way to improve health outcomes for patients, health service quality has drawn the attention of governments and managers [6].

Increasing studies from other service domains have proved that online WOM significantly impacts consumers’ purchase behaviors [7] and product sales [8, 9]. However, the effect of WOM review systems on patient choice in the health field remains under-explored. Service quality in existing studies is generally divided into two dimensions: technical quality and functional quality [10]. However, for other service domains, most of the studies on service quality focus primarily on functional quality (service delivery process) [11]. Only a very limited number of studies incorporate the technical quality (service outcome) [12]. Richard and Allaway [13] asserted that restricting explanations of consumer behaviors exclusively to functional quality is a misspecification of service quality. By way of contrast, there are few studies that investigate the role of service quality in the health service domain; those studies often excessively emphasize the importance of technical quality and tend to ignore the functional quality. As a result, this paper attempts to ascertain how patients choose physicians, based on cumulative WOMs from prior consumers about physicians’ technical quality *as well as* their functional quality.

In this paper, we empirically explored the impact of physicians’ WOM on patient choice using data from a Chinese health community. We studied a unique service: online booking and service in hospital (OBSH), which assists patients seeking to access hospital services. OBSH differs from e-commerce. Customers using OBSH do not need to receive physical products, while express delivery is universally expected by customers of e-commerce services. In light of the distinct characteristics of the health field, we integrated two dimensions into our study—technical quality and functional quality—to measure service quality in the health services [10]. We also examined how the interactions between these two dimensions affect patient choice. Moreover, we attempted to study how the impacts of different dimensions of service quality on patient choice vary, based on the patients’ characteristics (e.g. disease risk).

### Literature review and hypotheses

**Fig. 1:**
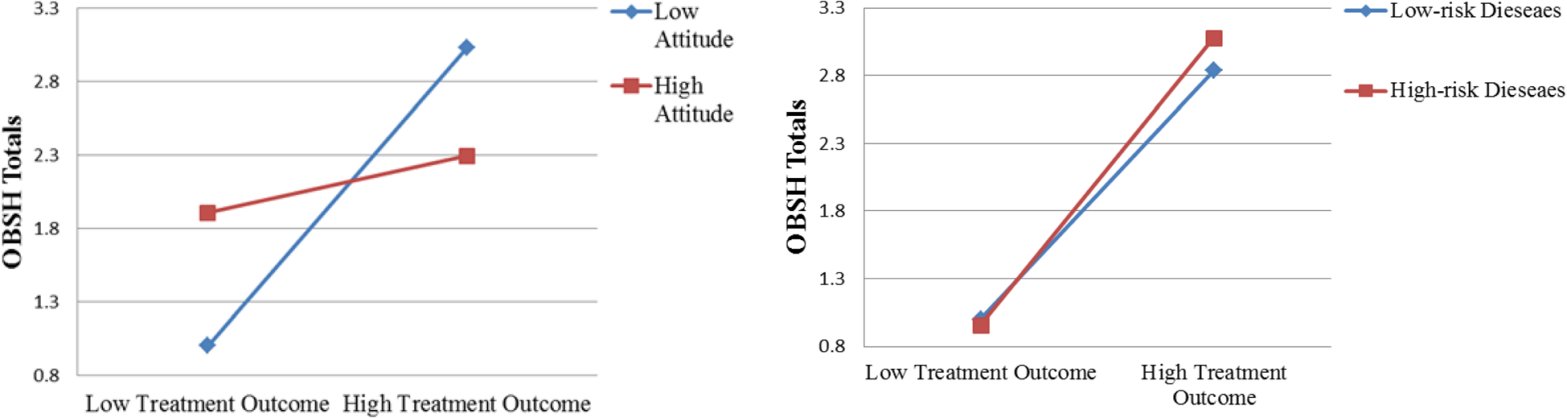
Conceptual Model

Service quality is a useful tool for customers to evaluate services in response to their experiences, and is, in turn, further utilized by government structures. Donabedian [14] supports our assertion that, like other service sectors, health service quality should also be divided into two dimensions: technical quality and functional quality. Technical quality is defined primarily on the accuracy of treatment and diagnoses, and functional quality refers to the manner in which the health service is delivered to the patients. Based on the concept of service quality, this study seeks to examine how patients choose physicians based on evaluations of service quality by other patients. We study how the interaction of different dimensions of service quality affects patient choice and how the risk of a patient’s disease moderates the relationship between service quality and patient choice. Our conceptual model is shown in Fig. 1, and we present all our hypotheses below.

### Service quality and patient choice

There is agreement in the existing literature that service outcomes significantly affect customer perceptions of service quality [15]. Technical quality (outcome quality) reflects the customer’s perception of the superiority of service experience [16]. Jamal and Naser [17] find that the core aspects of service quality are directly linked to customer satisfaction. Similarly, Hsieh and Hiang [18] demonstrated that the perceived technical quality of service positively influences customer satisfaction.

However, physicians need to pay attention to not only their technical quality, but also their functional quality. The aim of health care is to improve wellbeing, not merely to cure the disease. It cannot address patients’ medical concerns without also addressing their anxiety and fears relating to their diseases. So, technical outcomes (e.g. treatment outcomes) are only half the answer. Functional quality influences patients’ emotional health and patients’ perception of physicians’ medical skill [19, 20]. The ideal physician must have both technical quality and functional quality (empathetic, humane, respectful, and thorough [21].

Furthermore, in medical sociology literature, DiMatteo et al. [22] find that physicians’ socio-emotional behavior such as caring and openness to communication tend to play an important part in patients’ decision process. The most important aspects of patient satisfaction with physicians are patients’ feelings of being genuinely cared-for: the degree to which their physicians took time with them, explained things patiently and listened to them, and were accessible when needed. Good bedside manner of physicians is highly valued by patients, despite having received little attention [23].

Based on above comments, we suppose there are positive effects arising due to the technical qualities and functional qualities on patient choice. So we develop the following hypotheses:

- Hypothesis 1a: *Higher technical quality of physician leads to more frequent selection of that physician*.
- Hypothesis 1b: *Higher functional quality of physician leads to more frequent selection of that physician*.

### The interaction between functional quality and technical quality

The participation of consumers in the production of services implies that besides the service outcome, the process through which the service is delivered to consumers should also affect service quality perception. The earlier studies tend to suggest a cumulative effect of technical quality and functional quality on consumers’ behavior [16, 24]. Following on from this, authors have sought to examine whether there is interaction effect of the two types of service quality on consumer behavior, and the existing literature argues for both positive and negative moderation effects of functional quality on the relationship between technical quality and patient behavior.

On the one hand, fairness heuristic theory [25] describes as long as customers perceive the service delivery process as fair, they are likely to display a positive response to unfavorable outcome. Based on the fairness heuristic theory, when patients receive favorable functional quality of service, they tend to use the pleasure of the process as a substitute in the assessment of the physicians’ service quality, even if they are exposed to unfavorable technical quality. From this perspective, functional quality negatively moderates the relationship between technical quality and patient choice.

On the other hand, two-factor theory [26] provides the greatest effect on customers’ post-consumption behavior is attained under the favorable process-favorable outcome condition. Based on the two-factor theory, when patients receive favorable functional quality of service, this will magnify the effects of positive perception of outcome quality on patient choice. From this perspective, functional quality positively moderates the relationship between technical quality and patient choice.

According to the empirical findings in the study by [27], the theoretical arguments derived from the individual theories are ‘contextually’ valid. For example, the pattern of negative mitigation (predicted by fairness heuristic theory) appears in the uncertain outcome condition, whereas the pattern of positive enhancement (derived from two-factor theory) emerges in the certain outcome condition [27]. Although treatment outcomes in the health field are difficult to measure, our study focused on how patients made their choices based on the existing quality reviews from other patients. It is of note that they could get clear evaluations no matter how truthful these evaluations are. As we cannot get conclusion on whether the interaction effect is negative or positive from the prior theory, we refer to the method of [28], which they propose both negative and positive hypotheses for this situation. Therefore, we propose both negative and positive hypotheses for the moderation effect of functional quality on the relationship between technical quality and patient choice.

- Hypothesis 2a: *Functional quality of physicians negatively moderates the relationship between technical quality and patient choice*.
- Hypothesis 2b: *Functional quality of physicians positively moderates the relationship between technical quality and patient choice*.

### The moderation effects of disease risk

The psychological choice model discussed in Hansen [29] suggests that the effectiveness of an influencer (such as online WOM) is moderated by environmental and contextual factors (consumer and product characteristics), and the interactions among these variables eventually determine the response (purchase decisions). Consistent with this framework, several studies show that consumers’ use of different information sources indeed varies with consumers’ or products’ characteristics. For example, Zhu and Zhang [30] indicate that online WOM is more influential for less popular games and games whose players have greater Internet experience.

In the health field, patients’ behavior is also influenced by their characteristics, among which disease type is a very important influencer. Existing studies argue that individual characteristics of patients moderate their reactions to the physicians’ communication style [31, 32]. Caring actions and assertiveness of physicians do not have the same impact on all patients. For instance, caring actions have a stronger influence on patient satisfaction when the patients have less severe illness [31, 33]. With some patients, low caring behaviors seem even better than high caring behaviors. To illustrate, patients who are more anxious about their health status sometimes prefer physicians whom they perceive as sounding less positive, but more risk-aware and concerned [32].

When a patient chooses a physician, type of disease is affecting them will affect their choice. When a patient suffers from a high-risk disease, s/he is more worried and therefore hopes to find a physician with higher technical quality, rather than solely a physician with high caring behaviors. Conversely, patient cares more about functional quality of physicians when affected by relatively low-risk diseases, and caring behaviors make patient more satisfied. Hence, we develop the following hypotheses.

- Hypothesis 3a: *The disease risk positively moderates the relationship between technical quality and patient choice*.
- Hypothesis 3b: *The disease risk negatively moderates the relationship between functional quality and patient choice*.

## Methods

### The research context

We focus on a Chinese online health community, Guahao.com (http://www.guahao.com/), which provides online booking services for patients around the country. It is authorized by the China Health and Family Planning Committee in March, 2010, and is the biggest health booking website in China with over twenty-three million real-name registered users and a hundred thousand experts.

On the website, patients can get physicians’ information from all over the country, including their hospitals’ information, medical titles, education experience, and work experience. Guahao.com updates physicians’ real-time booking availability and displays this information on physicians’ homepages. Moreover, it provides an experience-sharing forum for patients who have already booked online and received service in those hospitals to share their treatment experiences. Patients can write their experiences after their illnesses have been cured or improved, and the website does not limit the time lag between patients’ receiving service and writing experiences. It helps to match the physicians’ services and patients’ demands accurately and effectively. An increasing number of patients use this website to find the right physicians and make appointments with them.

Guahao.com provides several features that fit with our study. First, Guahao.com constructs a formal and comprehensive feedback mechanism for patients to express their evaluation on service quality of physicians, and patients can get that information and make choices accordingly. Second, Guahao.com is the largest health community that provides appointment function specifically for people. It has great many users and brings convenience for them. Based on the above features, Guahao.com is a fundamentally useful website for our study.

### Sample and data collection

Like other OHCs website, Guahao.com divides all physicians based on their departments and diseases. According to the Chinese Health Statistics Yearbook in 2013 [34], which lists the mortality rate of various diseases, we chose the most fatal category of diseases from 2012: malignant tumor-related diseases, and randomly selected 8 diseases from this class of disease as high-risk diseases. We randomly selected another category of diseases as low-risk diseases: endocrine related diseases. We then randomly selected 8 diseases from another category of diseases in the list of the Yearbook as low-risk diseases. The difference in the mortality rates of the two types of diseases is significant enough for us to distinguish between them and use them to represent high-risk diseases and low-risk diseases respectively (mortality rate of malignant tumor related diseases is nearly 164.51/100,000 in 2013, rank 1; mortality rate of endocrine related diseases is nearly 17.32/100,000 in 2013, rank 7). Following this procedure, 16 diseases were ultimately included in our study, including 8 high-risk diseases and 8 relatively low-risk diseases. We collected physicians’ information and patients’ information data from the platform, and this process was repeated once after one month in order to get the variation of the dependent variable. Finally, we had collected 1,853 physicians’ information. For the physicians in our dataset, we collect their service information, WOM information, and information of the hospital and the department that the physicians belong to.

### Variables and empirical models

We used OLS model to test our hypotheses. Our empirical variables are shown in Table 1. As we examine the specific service which is booked online and received in hospital (OBSH), so our dependent variable is the number of OBSH for each physician.

**Table 1:** Variables description

Variables		Variables symbol	Explanation
Dependent variable	Patients appointment totals	*LnOBSH_Totals*	The number of patient appointments; we use the log value.
Independent variables	Physician treatment outcome	*Treatment*_*outcome*	Patients give an evaluation score for physicians’ technical quality when patients share experiences.
	Physician’s attitude	*Attitude*	Patients give an evaluation score for physicians’ functional quality when patients share experience.
	Disease type	*Disease*_*dummy*	The disease risk that patients get. We use one dummy variable to measure the disease risk. When the disease is high-risk, the variable equal to 1.
Control variables	Hospital level	*Hlevel*_*dummy1,*	Hospital has their own levels, which is judged by the country and can represent their ability: level 1, 2 and 3. Level 1 is the best. Some hospitals do not have a level, because their medical abilities do not reach the level 3, so we use three dummy variables to represent level 1, 2, 3, and no level.
		*Hlevel*_*dummy2*,	
		*Hlevel*_*dummy3*	
	Hospital score	*H*_*Score*	When patients write experiences, they also give a score on the hospital’s environment and attitude of guide service.
	Physician titles	*Title*_*dummy1*,	Physicians have their own titles, which is judged by the country, and can represent their medical skill. These titles include chief physician, associate chief physician, attending physician and other titles. The number of other title physicians is small, so we use two dummy variables to represent chief physician and associate chief physician.
		*Title*_*dummy2*	
	Physician experience totals	*LnExperience Totals*	The number of patient experiences for the physician; we use the log value.

For service quality measures, Guahao.com provides two separate feedbacks on technical quality and functional quality. When a patient has finished using the OBSH service, they can choose to write about their experiences on the platform. If a patient does so, they need to give two scores for perceived treatment outcome and perceived physician’s attitude separately. In this paper, we use perceived treatment outcome to measure the technical quality and perceived physician’s attitude to measure the functional quality. The website calculates the mean of perceived treatment outcome and the mean of physician’s attitude for each physician based on feedbacks from all patients of the physician, so we use the mean of perceived treatment outcome and the mean of physician’s attitude in our model.

Disease risk mainly refers to the mortality rate of diseases [35, 36]. In order to distinguish risks of different diseases, we choose diseases with significantly different mortality rates in our sample. According to the Chinese Health Statistics Yearbook in 2013 [34], which lists the mortality rate of various categories of diseases (see Table 2), we selected the most fatal category of diseases as our high-risk diseases: malignant tumor related diseases. We then randomly select 8 diseases from this category of diseases, including lung cancer, breast cancer, lymph cancer, gastric cancer, colon cancer, rectal cancer, nasopharynx cancer, and liver cancer. Those diseases represent our high-risk diseases (mortality rate is nearly 164.51/100,000 in 2013, rank 1). In the lists provided in the Chinese Health Statistics Yearbook in 2013, we randomly selected another category of diseases—endocrine related diseases—as low-risk diseases. We also randomly selected 8 diseases from this category, including diabetes, thyroiditis, endocrinopathy, osteoporosis, hyperthyroidism, hypothyroidism, hypoglycemia, and bradygenesis. Those diseases represent our relatively low-risk diseases (mortality rate is nearly 17.32/100,000 in 2013, rank 7). The difference in the mortality rates of these two categories of disease is large enough (nearly 10 times) to enable us to carry out this study. In our analysis, we introduced a dummy variable to represent the disease risk. When the disease risk is high, it equals to 1, otherwise equals to 0.

**Table 2:** Mortality Rate of Main Diseases

Disease Name	Mortality Rate (1/100,000)	Rank
Malignant tumor related diseases	164.51	1
Heart Diseases	131.64	2
Cerebrovascular related diseases	120.33	3
Respiratory related diseases	75.59	4
Injure and Intoxication	34.79	5
Other diseases	23.82	6
Endocrine, metabolic and nutritional related diseases	17.32	7
Digestive system diseases	15.25	8
Nervous system diseases	6.86	9
Urogenital diseases	6.30	10
…	…	…

We also used hospital information and other information of the physicians as the control variables in the model. For hospital information, we included the hospital level, which represents hospitals’ medical ability and is judged by the country as level 1 (best), 2, and 3 (worst). Some hospitals have no level, because their medical abilities do not reach level 3, so we used three dummy variables for hospital level. Besides, patients could potentially give a score for the environment of the hospital when he/she writes about an experience, so we also included this hospital score in our model. In addition, physicians have their own titles in the hospital, including chief physician, associate chief physician, attending physician and other. In our data set, we only have very few physicians without a title, so we used two dummy variables to measure the physician title. Finally, existing research shows a significant relationship between WOM totals and sales [37]. We also included the number of patient experiences in our model. Below specifies our dummy variables:

$$ \begin{array}{l} Hlevel\_ dummy1\left\{\begin{array}{l}=1,\ \mathrm{when}\ \mathrm{hospital}\ \mathrm{level}\ \mathrm{is}\ \mathrm{level}\ 1\\ {}=0,\kern1.5em \mathrm{others}\end{array}\right.\\ {} Hlevel\_ dummy2\left\{\begin{array}{l}=1,\ \mathrm{when}\ \mathrm{hospital}\ \mathrm{level}\ \mathrm{is}\ \mathrm{level}\ 2\\ {}=0,\kern1.5em \mathrm{others}\end{array}\right.\\ {} Hlevel\_ dummy3\left\{\begin{array}{l}=1,\ \mathrm{when}\ \mathrm{hospital}\ \mathrm{level}\ \mathrm{is}\ \mathrm{level}\ 3\\ {}=0,\kern1.5em \mathrm{others}\end{array}\right.\\ {} Title\_ dummy1\left\{\begin{array}{l}=1,\ \mathrm{when}\ \mathrm{doctor}\ \mathrm{title}\ \mathrm{is}\ \mathrm{chief}\ \mathrm{physician}\\ {}=0,\kern1.5em \mathrm{others}\end{array}\right.\\ {} Title\_ dummy2\left\{\begin{array}{l}=1,\ \mathrm{when}\ \mathrm{doctor}\ \mathrm{title}\ \mathrm{is}\ \mathrm{associate}\ \mathrm{chief}\ \mathrm{physician}\\ {}=0,\kern1.5em \mathrm{others}\end{array}\right.\end{array} $$

As we collected data from two time points separately for the dependent variable in the model, we used changes occurring between the two time points. All other variables in the model are measured by the data collected at the earlier time point. Our empirical model is shown as follows:

$$ \begin{array}{l} LnOBSH\_ Total{s}_t- LnOBSH\_{Totals}_{t-1}\\ {}\kern5em ={\beta}_{i1} Hlevel\_ dummy{1}_{t-1}+{\beta}_{i2} Hlevel\_ dummy{2}_{t-1}+{\beta}_{i3} Hlevel\_ dummy{3}_{t-1}\\ {}+{\beta}_{i4}H\_ Scor{e}_{t-1}+{\beta}_{i5} Title\_ dummy{1}_{t-1}+{\beta}_{i6} Title\_ dummy{2}_{t-1}\\ {}\kern5.25em +{\beta}_{i7} LnExperience\_ Total{s}_{t-1}+{\beta}_{i8} Treatment\_ outcom{e}_{t-1}+{\beta}_{i9} Attitud{e}_{t-1}+{\beta}_{i10} Disease\\ {}+{\beta}_{i11} Treatment\_ outcom{e}_{t-1}\times Attitud{e}_{t-1}\\ {}+{\beta}_{i12} Treatment\_ outcom{e}_{t-1}\times Disease+{\beta}_{i13} Attitud{e}_{t-1}\times Disease+\varepsilon \end{array} $$

## Results

Table 3 shows the descriptive statistics and correlations for key variables used in the empirical model. From Table 3, both treatment outcome (variable 9) and physician’s attitude (variable 10) have positive correlations with physician appointment amount (variable 1), and the beta coefficients are 0.724 and 0.623 respectively.

**Table 3:** Description and Correlation

	Mean	Std	1	2	3	4	5	6	7	8	9	10	11
*1. LnOBSH*_*Totals*	1.218	1.724	1										
*2. Hlevel*_*dummy1*	0.75	0.431	0.304^**^	1									
*3. Hlevel*_*dummy2*	0.09	0.284	−0.165^**^	−0.544^**^	1								
*4. Hlevel*_*dummy3*	0.09	0.285	−0.180^**^	−0.546^**^	−0.097^**^	1							
*5. H*_*score*	5.740	4.189	0.424^**^	0.501^**^	−0.271^**^	−0.341^**^	1						
*6. Title*_*dummy1*	0.35	0.477	0.253^**^	0.097^**^	−0.077^*^	−0.066	0.107^**^	1					
*7. Title*_*dummy2*	0.43	0.495	−0.073^**^	−0.003	0.087^*^	−0.003	−0.039^**^	−0.636^**^	1				
*8. LnExperience*_*Totals*	0.397	0.827	0.835^**^	0.205^**^	−0.129^**^	−0.123^**^	0.301^**^	0.235^**^	−0.097^**^	1			
*9. Treatment*_*outcome*	0.205	0.376	0.724^**^	0.264^**^	−0.156^**^	−0.161^**^	0.377^**^	0.218^**^	−0.052^*^	0.671^**^	1		
*10. Attitude*	0.479	0.476	0.623^**^	0.308^**^	−0.171^**^	−0.236^**^	0.500^**^	0.242^**^	−0.049^*^	0.425^**^	0.518^**^	1	
*11. Disease*_*dummy*	0.107	0.309	−0.176^**^	0.098	−0.041^*^	−0.023	0.015^*^	−0.053^**^	0.076^*^	−0.139^**^	−0.156^**^	−0.163^**^	1

Note: **p < 0.01, *p < 0.05

We used an OLS model to test our hypotheses, and the results are shown in Table 4. We present our result models hierarchically, such that we include all the control variables in model 1. We then introduced the independent variables and the interaction terms in model 2 and model 3, respectively. The adjusted R-square value and the significance of F-value suggest that our independent variables explain our dependent variable well. The variance inflation factor (VIF) values of all variables are below 1, which indicates the absence of multicollinearity. Moreover, we account for heteroskedasticity and report heteroskedasticity-consistent standard errors in all our models.

**Table 4:** Empirical Model Results

Variables	Model1	Model2	Model3
Hlevel_dummy1	0.352***	0.379***	0.363***
	(0.084)	(0.073)	(0.072)
Hlevel_dummy2	0.171*	0.272***	0.261***
	(0.105)	(0.090)	(0.089)
Hlevel_dummy3	0.147	0.285***	0.277***
	(0.104)	(0.090)	(0.089)
H_Score	0.065***	0.016***	0.016***
	(0.005)	(0.005)	(0.005)
Title_dummy1	0.344***	0.077	0.079
	(0.057)	(0.050)	(0.050)
Title_dummy2	0.219***	0.065	0.061
	(0.054)	(0.048)	(0.046)
LnExperience_Totals	1.582***	1.231***	1.221***
	(0.026)	(0.029)	(0.029)
Technical Quality			
Treatment_outcome		0.758***	3.502***
		(0.068)	(0.628)
Functional Quality			
Attitude		0.889***	0.952***
		(0.048)	(0.050)
Disease_dummy		−0.194***	−0.108
		(0.059)	(0.069)
Treatment_outcome* Attitude			−2.970***
			(0.659)
Treatment_outcome* Disease			1.155***
			(0.326)
Attitude*Disease			−0.473***
			(0.139)
Adjusted-R^2^	0.734	0.806	0.811
F change	743.903***	217.885***	13.714***

Note: ***p < 0.001, **p < 0.01, *p < 0.05, +*p* < 0.10

Hypothesis 1a and 1b test the impact of technical quality and functional quality on the number of patient choice. According to model 2 in Table 4, we found there is a significant and positive impact of treatment outcome on patient choice (*β* = 0.758, *p* < 0.001), and the physician’s attitude also significantly and positively affects patient choice (*β* = 0.889, *p* < 0.001). The results indicate that patients like to make appointments with physicians with high medical skill and good attitude, and our hypotheses 1a and 1b are both supported.

Hypotheses 2a and 2b examine the interaction effect of functional quality and technical quality on patient choice. From model 3 in Table 4, we found the interaction term is negative and significant (*β* = −2.970, *p* < 0.001), so hypothesis 2a is supported. Our results suggest that physician’s attitude negatively moderates the relationship between treatment outcome and patient choice.

Hypotheses 3a and 3b test the moderation effects of disease risk on the relationships between service quality and patient choice. Our results show that disease risk significantly and positively moderates the relationship between treatment outcome and patient choice (*β* = 1.155, *p* < 0.001). However, disease risk significantly and negatively moderates the relationship between the physician’s attitude and patient choice (*β* = −0.473, *p* < 0.001). Thus, our Hypotheses 3a and 3b are both supported.

**Fig. 2:**
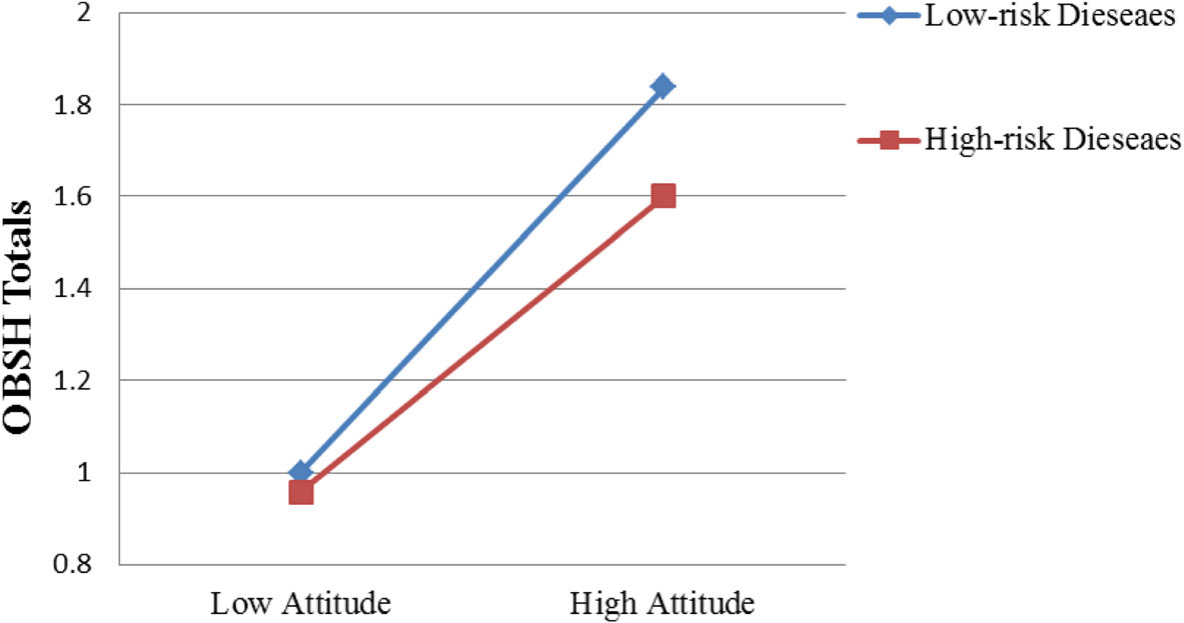
The Interaction Effects

The interaction effect and moderation effects are illustrated in Fig. 2. For the interaction effect of treatment outcome and attitude, and the moderation effects of disease risk on the relationship between service quality and OBSH, we use mean value of treatment outcome and attitude variable plus its standard deviation to present high treatment outcome and attitude, and mean value of treatment outcome and attitude minus its standard deviation to present low treatment outcome and attitude, and we found both value of low and high value (treatment outcome and attitude) located within the scope of them. See the following formula:

$$ \begin{array}{l}\mathrm{High}\ \mathrm{treatment}\ \mathrm{outcome}=\mathrm{mean}\ \mathrm{value}\ \left( Treatment\_ outcom{e}_{t-1}\right) + \mathrm{standard}\ \mathrm{deviation}\left( Treatment\_ outcom{e}_{t-1}\right)\kern0.5em \\ {}\mathrm{Low}\ \mathrm{treatment}\ \mathrm{outcome}=\mathrm{mean}\ \mathrm{value}\ \left( Treatment\_ outcom{e}_{t-1}\right) - \mathrm{standard}\ \mathrm{deviation}\ \left( Treatment\_ outcom{e}_{t-1}\right)\ \\ {}\mathrm{High}\ \mathrm{attitude}=\mathrm{mean}\ \mathrm{value}\ \left( Attitud{e}_{t-1}\right) + \mathrm{standard}\ \mathrm{deviation}\ \left( Attitud{e}_{t-1}\right)\kern0.5em \\ {}\mathrm{Low}\ \mathrm{attitude}=\mathrm{mean}\ \mathrm{value}\ \left( Attitud{e}_{t-1}\right) - \mathrm{standard}\ \mathrm{deviation}\ \left( Attitud{e}_{t-1}\right)\ \end{array} $$

Figure 2 shows that the effect of technical quality on patient choice is significant under both low functional quality and high functional quality, but the impact is stronger under the low functional quality than under the high functional quality. Meanwhile, the effect of technical quality is also significant under both low disease risk and high disease risk, but the impact is stronger under high disease risk than under low disease risk. On the contrary, the effect of functional quality is significant for both low-risk diseases and high-risk diseases, but the impact is stronger for low-risk diseases than for high-risk diseases.

### Robustness check

In order to check the robustness of our model, we divided the main sample into two sub-samples, for high-risk diseases and relatively low-risk diseases respectively. In this part, we conducted one regression for high-risk diseases and one regression for low-risk diseases (with no interaction with disease risk). We wanted to check whether the main effects (H1a-H2b) still hold in the sub-samples.

We use the same symbols in the model and results are shown in Table 5. From the robustness check results, we still observe that technical quality and functional quality both positively impact patient choice and their interaction term negatively impact patient choice. All the main effects are consistent with the results using the whole sample.

**Table 5:** Empirical Model Results (robust check)

Variables	High-risk Diseases	Low-risk Diseases
Hlevel_dummy1	0.009	0.367**
	(0.126)	(0.075)
Hlevel_dummy2	0.009	0.255**
	(0.166)	(0.094)
Hlevel_dummy3	-	0.270**
	-	(0.094)
H_Score	0.006	0.016*
	(0.011)	(0.005)
Title_dummy1	0.180*	0.056
	(0.100)	(0.054)
Title_dummy2	0.092	0.048
	(0.081)	(0.051)
LnExperience_Totals	0.274	1.220***
	(0.210)	(0.030)
Technical Quality		
Treatment_outcome	10.472***	3.488***
	(2.465)	(0.660)
Functional Quality		
Attitude	0.550***	0.953***
	(0.097)	(0.053)
Treatment_outcome* Attitude	−8.968***	−2.952***
	(2.626)	(0.694)
Adjusted-R^2^	0.770	0.807
F	F(9,106) = 43.95	F(10,1643) = 695.57

Note: ***p < 0.001, **p < 0.01, *p < 0.05, +*p* < 0.10.

## Discussion and conclusions

Our findings provide us with valuable insight into the role of service quality in the health field. Specifically, hypotheses 1a and 1b examine the impact of two dimensions of service quality on physicians’ OBSH totals in the future. Hypotheses 2a and 2b investigate the impact of the interaction between functional quality and technical quality on physicians’ OBSH totals. Finally, hypotheses 3a and 3b study the moderation effect of disease risk on the relationships between service quality and patient choice. From our empirical results and robustness check results, all the hypotheses are supported.

Overall, our statistical evidence suggests that physicians with high technical quality and functional quality are more likely to attract patients in the future. Besides, we prove that functional quality negatively moderates the relationship between technical quality and patient choice under our research context. Moreover, the disease risk moderates the relationship between physician service quality and patient choice. Specifically, for high-risk diseases, patients care more about technical quality than they do for low-risk diseases, and for low-risk diseases, patients care more about functional quality than high-risk diseases.

Our results suggest that in the health field, like consumers in other fields, patients are very concerned about the quality of service. Our empirical results are consistent with other studies on service quality, where service quality is an important factor that influences the consumer choice. Our results suggest that better service of physicians leads to a positive behavioral intention and increases the purchase intention of the patients.

Furthermore, our empirical results state that there is a substitution effect between functional quality and technical quality within our research context. As patients are paying more and more attentions to physicians’ attitudes, technical solutions are only half the answer, and physicians’ behavior tends to weigh heavily in patients’ decisions [22]. Patients care about emotional support, which makes them feel that they are being cared-for and respected, and this gives them confidence to combat their diseases. When patients experience an unfavorable treatment outcome, the perception of service process may function as a compensation for that outcome and significantly affect their reactions [25]. Hence, our results show that physicians should increasingly pay attention to their behaviors and attitudes.

Moreover, our empirical results show that patients’ choices are influenced by their situation by the level of risk associated with their disease. Patients with different health problems value different dimensions of service quality. Patients who suffer from high-risk diseases bear both physical and psychological pressures. They hope to have their diseases treated comprehensively and promptly, and prefer that physicians be more serious and treat them as well as possible [32]. These patients with high-risk diseases are influenced by previous treatment outcomes of physicians more strongly than patients with low-risk diseases. On the contrary, the attitudes of physicians have a stronger influence on patients with low-risk diseases than high-risk diseases.

The findings of this study present significant theoretical implications. Firstly, our study fills existing research gaps on service quality by investigating the impact of service quality on a specific service that makes choices online and receives services offline. Prior studies have proven that better service quality leads to positive behavioral intention [38], however, they rarely examine both technical quality and functional quality simultaneously. Studies in the other service industries mainly focus on functional quality, and studies in the health field mainly focus on medical skills (technical quality). However, examining only one dimension to explain consumers’ behavior may lead to a misspecification of service quality [13]. Patients feel both physical and mental pain. They are eager to be cured, and also need emotional support, because they are vulnerable. Therefore, these two dimensions are both very important, and neither can be ignored.

Secondly, our study examines the interacting effect of functional quality and technical quality in the health field. Existing studies argue for both complementary and substitution effects [25, 26], and we suggest that the impact of functional quality substitutes the impact of technical quality on patients’ behaviors in the health field. Our study reveals that it is not only the treatment outcome that is important, but also the attitude of physicians. Even if a doctor has low technical quality, patients will also like to choose him if he has a high functional quality. Our study enriches existing studies on the interaction of functional quality and technical quality in the health field, and empirically confirms the substitution effect.

Thirdly, we enrich existing studies on the moderating effect of individual characteristics on consumers’ choices in the health field by demonstrating the moderating effect of disease risk on the relationship between service quality and patient choice. Health related factors need to be taken into account when studying patient choice, as patients’ behaviors are primarily dominated by theses individual characteristics [39]. However, this area remains less investigated, and our results show that how patients’ decision relies on technical quality (perceived treatment outcome) and functional quality (perceived physician’s attitude) differs according to the level of risk presented by the disease being treated.

This study also has significant practical implications. For physicians, our findings suggest that patient choice is not only positively influenced by technical quality but also positively influenced by functional quality, and that there is also a substitution effect between functional quality and technical quality. With developments within the health field, patients care about the human, emotional concern of physicians in addition to their medical skills. Therefore, it is recommended that physicians need to pay attention to both medical skill and service attitude and attempt to achieve a balance. Moreover, physicians also need to achieve a balance between technical quality and functional quality according to the seriousness and type of disease that they are treating. If a physician treats high-risk diseases, they should pay particular attention to improvements of their medical skills; contrastingly, if a physician treats low-risk diseases, they may have scope to pay extra attention to improve their clinical manner.

Furthermore, managers of hospitals need to consider the roles of both technical quality and functional quality seriously. With the parallel development of economies and medical techniques, patients’ demands on health care are increasing constantly, especially their demands of compassionate and sensitive care. Managers should not only pay attention to the improvement of the medical skill of physicians, and they also need to improve the attitudes and clinical manner of physicians. For example, by training physicians regularly, and bringing physician’s clinical manner into physicians’ performance assessments is helpful.

Nonetheless, this paper has its own limitations, which opens opportunities for future research. Firstly, we chose diseases based on the mortality rates of different categories of disease, but we did not investigate the mortality rate for every specific disease. Although the difference in mortality rates of the chosen two categories of diseases is quite large (about ten times), future research should select diseases more meticulously. Secondly, we just studied one context—the “online booking and service in hospital” (OBSH). This helps us improve the internal validity, but it may also reduce the broader applicability of our findings. Future research should validate our results in other service contexts. Thirdly, due to the limitation of data, we only conducted a cross-sectional analysis. Future research can adopt a longitudinal perspective to uncover the dynamics in the relationships as well.

## Abbreviations

OBSH: Online booking and service in hospital
OHCs: Online health communities
OLSm: Ordinary least squares
WOM: Word-of-Mouth
